# A Comparison of Single- and Multiparametric MRI Models for Differentiation of Recurrent Glioblastoma from Treatment-Related Change

**DOI:** 10.3390/diagnostics11122281

**Published:** 2021-12-06

**Authors:** Felix Eisenhut, Tobias Engelhorn, Soheil Arinrad, Sebastian Brandner, Roland Coras, Florian Putz, Rainer Fietkau, Arnd Doerfler, Manuel A. Schmidt

**Affiliations:** 1 Department of Neuroradiology, University Hospital Erlangen, Schwabachanlage 6, 91054 Erlangen, Germany; tobias.engelhorn@uk-erlangen.de (T.E.); arnd.doerfler@uk-erlangen.de (A.D.); manuel.schmidt@uk-erlangen.de (M.A.S.); 2 Department of Neurosurgery, University Hospital Erlangen, Schwabachanlage 6, 91054 Erlangen, Germany; soheil.arinrad@uk-erlangen.de (S.A.); sebastian.brandner@uk-erlangen.de (S.B.); 3 Department of Neuropathology, University Hospital Erlangen, Schwabachanlage 6, 91054 Erlangen, Germany; roland.coras@uk-erlangen.de; 4 Department of Radiation Oncology, University Hospital Erlangen, Universitaetsstrasse 27, 91054 Erlangen, Germany; florian.putz@uk-erlangen.de (F.P.); rainer.fietkau@uk-erlangen.de (R.F.)

**Keywords:** glioblastoma, cerebral radiation necrosis, pseudoprogression, treatment-related changes, dynamic susceptibility contrast perfusion imaging, apparent diffusion coefficient, multivariate logistic regression

## Abstract

To evaluate single- and multiparametric MRI models to differentiate recurrent glioblastoma (GBM) and treatment-related changes (TRC) in clinical routine imaging. Selective and unselective apparent diffusion coefficient (ADC) and minimum, mean, and maximum cerebral blood volume (CBV) measurements in the lesion were performed. Minimum, mean, and maximum ratios_CBV_ (CBV_lesion_ to CBV_healthy white matter_) were computed. All data were tested for lesion discrimination. A multiparametric model was compiled via multiple logistic regression using data demonstrating significant difference between GBM and TRC and tested for its diagnostic strength in an independent patient cohort. A total of 34 patients (17 patients with recurrent GBM and 17 patients with TRC) were included. ADC measurements showed no significant difference between both entities. All CBV and ratios_CBV_ measurements were significantly higher in patients with recurrent GBM than TRC. A minimum CBV of 8.5, mean CBV of 116.5, maximum CBV of 327 and ratio_CBV_ _minimum_ of 0.17, ratio_CBV_ _mean_ of 2.26 and ratio_CBV_ _maximum_ of 3.82 were computed as optimal cut-off values. By integrating these parameters in a multiparametric model and testing it in an independent patient cohort, 9 of 10 patients, i.e., 90%, were classified correctly. The multiparametric model further improves radiological discrimination of GBM from TRC in comparison to single-parameter approaches and enables reliable identification of recurrent tumors.

## 1. Introduction

Histologically characterized by high mitosis, necrosis, and diffuse infiltration of the surrounding brain [1,2], glioblastoma (GBM) resembles the most aggressive malignant primary intracranial neoplasm [3]. Despite the introduction of tumor-treating fields and the vascular epithelial growth factor (VEGF) inhibitor bevacizumab [4,5] to the standard approach of radical resection followed by concomitant radiochemotherapy [6,7] and significant advancements regarding guided surgical resection including the application of 5-aminolevulinic acid and contrast-enhanced ultrasound [8], this glioma subtype still marks a therapeutical challenge [9,10,11,12,13] and has the poorest prognosis with a median survival of under two years [14]. Furthermore, its high recurrence rate [15] limiting the patient’s survival represents a radiologic problem: Innumerable studies tried to differentiate between recurrent tumors and treatment related changes [16,17,18,19,20,21,22,23,24]—a question with the highest clinical impact [25,26] and a neuroradiological challenge. Whereas initial diagnosis of GBM is often reliably feasible due to its typical radiological features, e.g., peripheral irregular ring enhancement, intralesional hemorrhage, and central necrosis [7,27,28], imaging of recurrent and/or progressive residual high-grade GBM is similar to therapy associated cerebral radiation necrosis (increasing contrast enhancement and progressive peritumoral edema at least six months up to several years after radiotherapy [29], often progresses without treatment) or pseudoprogression (increasing contrast enhancement and progressive peritumoral edema within three to six months following radiotherapy, often resolves spontaneously) after surgical resection and concomitant radiochemotherapy; both show strong contrast enhancement, increasing fluid-attenuated inversion recovery (FLAIR) hyperintensities adjacent to the enhancement, and present with punctiform intralesional hemorrhage and necrosis [29,30].

The major drawback common to most of the previously proposed radiologic methods aiming for radiologic differentiation of GBM and TRC and thus impending their application in clinical routine is their complex, tedious postprocessing and the necessity of third-party software.

Hence, in this study, we wanted to test single- and multiparametric MRI from the clinical routine for differentiation of recurrent GBM and TRC. First, we evaluated selective and unselective apparent diffusion coefficient (ADC) measurements as well as dynamic susceptibility contrast (DSC) perfusion imaging in single parameter approaches and compared our diagnostic accuracy to previous studies. Second, we computed and evaluated a multiparametric model for fast and easy radiologic identification of patients with recurrent GBM in the clinical routine.

## 2. Materials and Methods

### 2.1. Patients

In retrospective, patients (from 2006 to 2019) with histologically diagnosed glioblastoma after radical surgical resection followed by radiotherapy and concomitant chemotherapy after the Stupp protocol [6] and continuous, in-house, pre- and post-operative, multiparametric 1.5 or 3 T follow-up MRIs were searched for in our interinstitutional tumor board. Only patients with a new contrast-enhancing lesion after completion of radiotherapy were considered for inclusion.

To validate the multiparametric model, it was tested for its diagnostic strength in an independent patient cohort with recurrent glioblastoma or TRC (from February to September 2021).

Written informed consent for MR imaging was obtained from all patients. The study was performed according to the Declaration of Helsinki and the European Guidelines for Good Clinical Practice. Additional ethical review was not required for participation in this retrospective analysis in accordance with local legislation (BayKrG Art. 27 (4)) and institutional requirements.

### 2.2. Acquisition and Postprocessing

#### 2.2.1. MRI

MRI examinations were performed at the department of neuroradiology of our hospital on a Magnetom Aera 1.5 T or on a Magnetom TrioTim 3 T (both Siemens Healthineers AG., Erlangen, Germany).

The first MRI dataset displaying the new contrast-enhancing lesion after completion of radiotherapy and concomitant chemotherapy was analyzed.

#### 2.2.2. Imaging Protocol and Sequence Details

All performed MRI examinations included a FLAIR sequence, a native T1 weighted sequence, a diffusion weighted imaging (DWI) sequence with ADC maps, DSC perfusion imaging with leakage correction and an isotropic contrast-enhanced T1 MP-RAGE sequence. In total, 50 measurements in a 2 s interval were performed for DSC perfusion imaging. At the fourth time point of DSC perfusion imaging, weight-adapted (0.2 mL/kg body weight) 0.5 mmol/mL DOTAREM (Guerbet, Villepinte, France) or DOTAGRAF (Jenapharm GmbH & Co. KG., Jena, Germany) was intravenously administrated at a flowrate of 3.5 mL/s, followed by a 20 mL saline flush. For further details of selected sequences, see Table 1.

#### 2.2.3. Postprocessing

Postprocessing of MRI datasets was performed in a standardized manner using the MR neurology workflow of a commercially available postprocessing software (syngo.via, Siemens Healthineers AG., Erlangen, Germany) including automatic computation of a local arterial input function (AIF) with manual plausibility check, a T1 leakage correction for DSC perfusion and automatic co-registration of the contrast-enhanced T1 MP-RAGE images and ADC and DSC datasets by the implemented linear registration tool.

**Figure 1:**
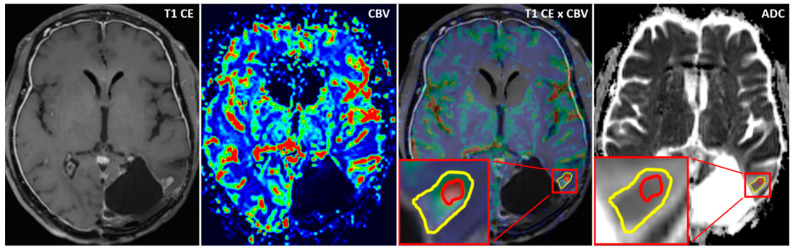
Exemplary cerebral blood volume and apparent diffusion coefficient measurements in a patient with recurrent glioblastoma. Exemplary apparent diffusion coefficient (ADC) measurement of a selective region of interest (ROI) (red) in the contrast-enhanced area with the highest cerebral blood volume (CBV) and an unselective ROI (yellow) comprising the whole contrast-enhancing lesion in a patient with recurrent glioblastoma (GBM). After placing the ROIs carefully in the fused CBV x contrast-enhanced T1 sequence map, ROIs are copied to the exact same position in the corresponding ADC map.

**Figure 2:**
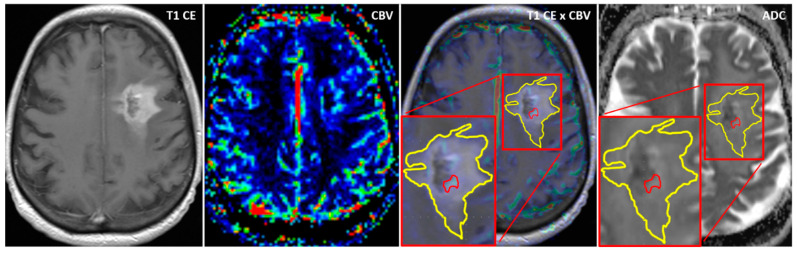
Exemplary cerebral blood volume and apparent diffusion coefficient measurements in a patient with treatment-related changes. Exemplary ADC measurement of a selective ROI (red) in the contrast-enhanced area with the highest CBV and an unselective ROI (yellow) comprising the whole contrast-enhancing lesion in a patient with treatment-related changes (TRC). After placing the ROIs carefully in the fused CBV x contrast-enhanced T1 sequence map, ROIs are copied to the exact same position in the corresponding ADC map.

Using the MCEval ROI (region of interest) tool of the mean curve plug-in of MR neurology, two ROIs were manually placed by a neuroradiologist (authors F.E. and M.A.S.) on the image slice depicting the largest lesion diameter in the co-registered MPRAGE x DSC map: a selective ROI in the contrast-enhancing lesion area with the highest CBV value excluding vessels and nontumorous tissue, and an unselective ROI contouring the whole contrast enhancing lesion including necrotic or cystic areas. Then, both ROIs were copied onto the corresponding ADC map. A third ROI was placed in the co-registered CBV perfusion and ADC maps in the contralateral healthy white matter excluding vessels, cerebrospinal fluid, and bone. Figure 1 and Figure 2 show exemplary measurements in a patient with recurrent GBM and a patient with TRC. Image post-processing and manual ROI placement took approximately 4 min.

### 2.3. Statistical Analysis

ADC and CBV values were analyzed using descriptive statistics and tested for normal distribution by using the D’Agostino–Pearson test (if *p* > 0.05, normality was accepted). The ratio of minimum, mean, and maximum CBV in the contrast enhancing area to minimum, mean, and maximum CBV in the contralateral, healthy white matter (ratio_CBV minimum/mean/maximum_) was computed for both recurrent GBM and TRC.

Then all values were tested for a significant difference between recurrent GBM and TRC via an unpaired, two-tailed *t*-test (if values showed a Gaussian distribution) or via the Mann–Whitney–U test (if values showed no Gaussian distribution).

A receiver operating characteristics (ROC) analysis was performed for selective and unselective minimum, mean and maximum ADC and CBV values as well as all CBV ratios using the Brown/Wilson hybrid method.

For multiparametric analysis, a multiple logistic regression analysis was performed using all significant, different CBV and ADC parameters to differentiate GBM and TRC.

Statistical analysis was performed with GraphPad Prism 8 (GraphPad Software, San Diego, CA, USA) and Excel (Microsoft, Redmond, WA, USA). Multiple logistic regression analysis was performed with SPSS 19 (IBM, Armonk, NY, USA). *p*-values less than 0.05 were considered statistically significant. P-values less than 0.05 are marked with “*”, less than 0.01 with “**”, less than 0.001 with “***” and less than 0.0001 with “****”.

## 3. Results

### 3.1. Patients

A total of 75 GBM patients with a new contrast-enhancing lesion after initial radical surgical resection followed by radiotherapy and concomitant chemotherapy of histologically proven GBM were introduced in our interinstitutional tumor board. A total of 34 patients (24 males, 10 females; mean age 62.2) received continuous pre- and post-operative, in-house, multimodal MR imaging and were included in our study. In 17 patients (14 males, 3 females; mean age 61.8) recurrent GBM was histologically proven via biopsy in accordance to the 2016 WHO classification [31]. In 17 patients (10 males, 7 females; mean age 62.6), TRC was diagnosed either via biopsy (*n* = 3) or based on the patient’s clinical symptoms (no significant clinical decline unrelated to comorbid event or concurrent medication) in combination with the further, short-term MRI follow-ups (*n* = 14) according to iRANO criteria [32]. Mean MRI follow-up time in the TRC cohort was 13.1 months to exclude recurrent tumors. Median time from radiotherapy to the occurrence of new contrast-enhancing lesions was 17.1 months for GBM and 20.8 months for TRC (see also Table 2).

In 15 of 17 patients with recurrent GBM, resection status was R0; in 2 patients with recurrent GBM, resection status was R2. In 15 of 17 patients with TRC, resection status was R0; in 2 patients with TRC, resection status was R2. 14 of 17 patients with recurrent GBM had no isocitrate dehydrogenase 1 (IDH1) mutation. In the other patients with recurrent GBM, IDH1 status was unknown. Furthermore, 8 of 17 patients with TRC had no IDH1 mutation, 4 of 17 patients with TRC had IDH1 mutation; in the other patients with TRC, IDH 1 status was unknown. Next, 3 of 17 patients with recurrent GBM showed a O6-methylguanine-DNA methyltransferase (MGMT) promoter methylation, 3 of 17 patients with recurrent GBM had no MGMT promoter methylation, and in the other patients with recurrent GBM, MGMT promoter methylation status was unknown. Finally, 3 of 17 patients with TRC showed a MGMT promoter methylation, 1 of 17 patients with TRC had no MGMT promoter methylation, and in the other patients with TRC, MGMT promoter methylation status was unknown.

### 3.2. ADC Results

**Figure 3:**
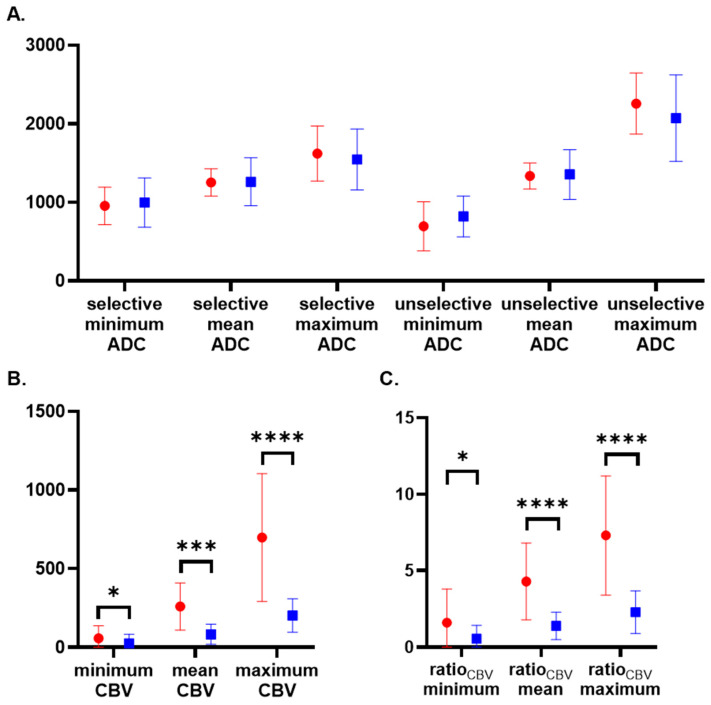
Apparent diffusion coefficient and cerebral blood volume measurements for differentiation of recurrent glioblastoma and treatment-related changes. Differentiation of recurrent GBM (red dot) and TRC (blue square) with both selective and unselective minimum, mean, and maximum ADC (**A**), minimum, mean, and maximum CBV (**B**) and minimum, mean, and maximum ratio_CBV_ (**C**). There was no statistically significant difference for ADC measurements. All CBV values and ratios were significantly higher in recurrent GBM than TRC. P-values less than 0.05 are marked with “*”, less than 0.001 with “***” and less than 0.0001 with “****”.

Neither selective nor unselective ADC values showed a significant difference between patients with GBM and TRC (see also Table 3 and Figure 3A).

### 3.3. CBV Results

Minimum, mean, and maximum CBV lesion values and the CBV ratios were significantly higher in patients with GBM than in patients with TRC (see also Table 4 and Figure 3B,C). CBV values of the unaffected contralateral white matter showed no significant difference between GBM and TRC.

**Figure 4:**
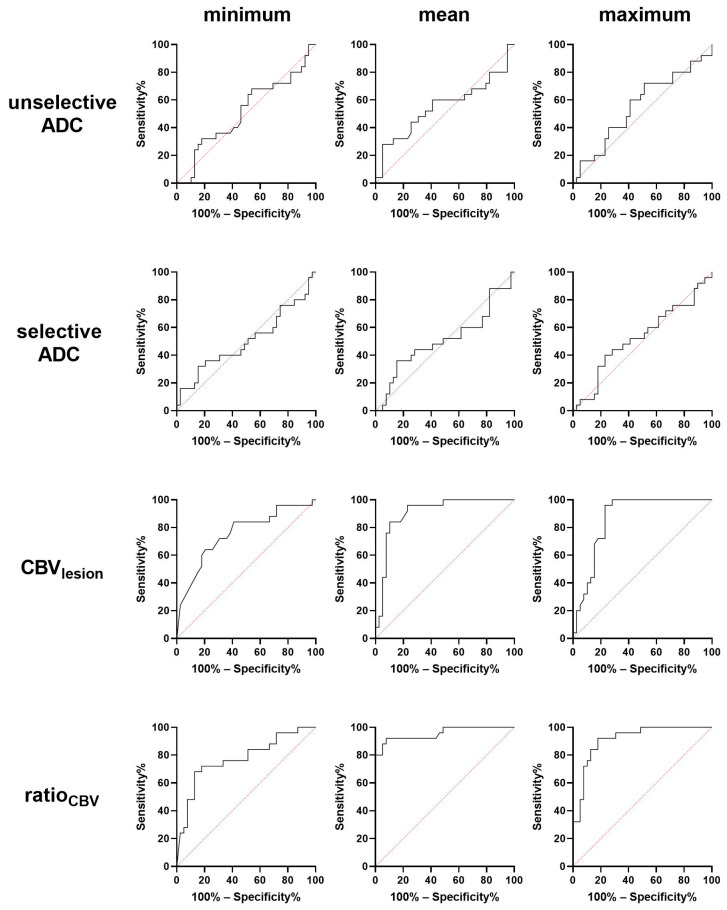
Receiver operating characteristic analysis of minimum, mean, and maximum apparent diffusion coefficient, cerebral blood volume, and ratio_CBV_ for differentiation of recurrent glioblastoma and treatment-related changes. Mean and maximum CBV and ratio_CBV mean_ and ratio_CBV maximum_ showed the highest area under the curve (AUC) for differentiation of both entities.

Using the AUC curves (see also Figure 4), the optimal cut-off values to differentiate GBM from TRC were determined (CBV_minimum_ = 8.5; CBV_mean_ = 116.5; CBV_maximum_ = 327; ratio_CBV minimum_ = 0.17; ratio_CBV mean_ = 2.26; ratio_CBV maximum_ = 3.82). This corresponds to the histological diagnosis in 71% of patients with recurrent GBM and 65% of patients with TRC for CBV_minimum_, in 82% of patients with recurrent GBM and 82% of patients with TRC for CBV_mean_, and in 82% of patients with recurrent GBM and 88% of patients with TRC for CBV_maximum_. This corresponds to the histological diagnosis in 88% of patients with recurrent GBM and 59% of patients with TRC for ratio_CBV minimum_, in 88% of patients with recurrent GBM and 82% of patients with TRC for ratio_CBV mean_, and in 88% of patients with recurrent GBM and 88% of patients with TRC for ratio_CBV maximum_.

### 3.4. Multiparametric Assessment

For multiparametric assessment minimum (odds ratio (OR) = 0.997, 95% confidence interval (CI) = 0.962 to 1.033), mean (OR = 1.007, 95% CI = 0.958 to 1.058), maximum CBV (OR = 1.002, 95% CI = 0.984 to 1.020), and ratio_CBV minimum_ (OR = 0.859, 95% CI = 0.574 to 1.286), ratio_CBV mean_ (OR = 4.485, 95% CI = 0.391 to 51.491), and ratio_CBV maximum_ (OR = 1.160, 95% CI = 0.287 to 4.692) as parameters with significant difference between GBM and TRC were used: Applying this model (Cox and Snell r-square = 0.555; Nagelkerkes r-square = 0.740; Hosmer–Lemeshow test = 0.625) further increased the probability for identification of recurrent GBM compared to the single parameter analysis; 94% of patients with recurrent GBM were classified correctly.

**Figure 5:**
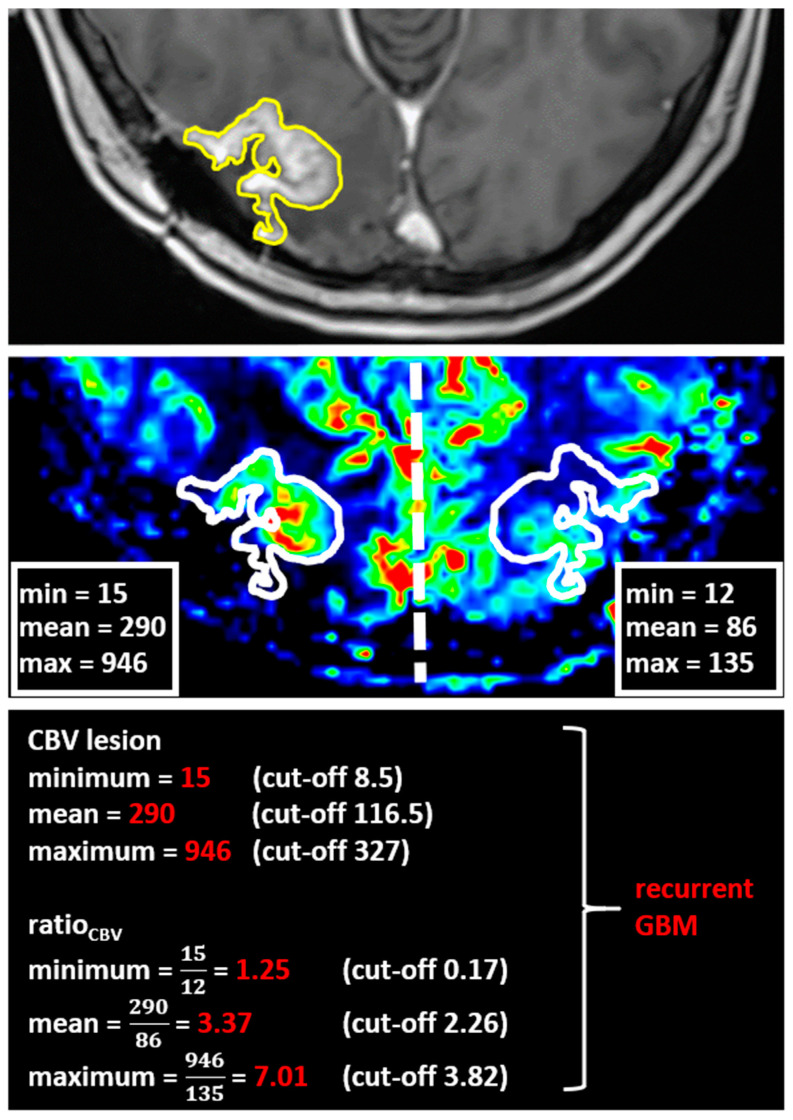
Exemplary application of the computed cerebral blood volume thresholds in the clinical routine to differentiate recurrent glioblastoma and treatment-related changes. A 60-year-old patient with a new contrast-enhancing lesion in the right occipital lobe (upper line) after radical resection and combined radiochemotherapy of a GBM. First, the whole contrast-enhancing lesion is contoured, then copied to the exact same position in the corresponding CBV map (middle). A second ROI is placed in the contralateral healthy hemisphere mirrored along the midline (falx cerebri). Applying our algorithm, we concluded the new lesion to be recurrent GBM (basal line). A biopsy confirmed the radiologic diagnosis.

Table 5 summarizes the results of the GBM and TRC classification for both the single-parameter approaches and the multiparametric model. Figure 5 shows an exemplary application of our multiparametric model for differentiation of GBM and TRC in one of our included patients in the clinical routine.

### 3.5. Testing the Multiparametric Model

To validate the multiparametric model, we tested it in an independent patient cohort with either recurrent glioblastoma (*n* = 5; 3 females, 2 males; mean age = 52.6) or TRC (*n* = 5; 4 males, 1 female; mean age = 57.4). In this cohort, the multiparametric model allowed correct identification of 100% recurrent GBM (5 of 5 patients) and 80% TRC (4 of 5 patients) resulting in 90% correct classifications.

## 4. Discussion

This study evaluated the diagnostic power of multiparametric MRI for differentiation of patients with recurrent GBM and TRC using DSC CBV perfusion and two different methods of ADC measurements—first the unselective contouring of the whole contrast-enhancing lesion, second by selective ROI placement in the contrast-enhancing area with the highest CBV value. Yet, neither unselective nor selective ADC minimum, mean, or maximum values showed a significant difference in recurrent GBM and TRC. In contrast, minimum, mean, maximum CBV and the minimum, mean, and maximum ratio_CBV_ between contrast-enhancing lesion and contralateral, healthy white matter showed significantly higher values in GBM than in TRC. Optimal cut-off values were computed to discriminate both groups (CBV_minimum_ = 8.5; CBV_mean_ = 116.5; CBV_maximum_ = 327; ratio_CBV minimum_ = 0.17; ratio_CBV mean_ = 2.26; ratio_CBV maximum_ = 3.82). The combination of these six parameters in a multiparametric model surpassed all single-parameter approaches in the identification of recurrent GBM allowing the correct radiological classification of recurrent tumors in 94% of the included patients. Furthermore, when testing the multiparametric model in an independent test cohort, 90% of included patients with either recurrent GBM or TRC were correctly identified.

Imaging-based differentiation of recurrent GBM and TRC is of great relevance for both patients and clinicians because of the severe difference in prognosis and therapeutic approaches [25,26]. Thus, several previous studies have tested multiparametric MRI for discrimination of these entities with a wide variety of different sequences, cut-off values, and ratios. However, the described approaches have still not made it into routine clinical imaging—although, most methods are commonly applied at least in neuroradiological centers. In a recent meta-analysis, van Dijken et al. evaluated a total of 35 studies based on anatomical parameters, ADC, DSC and DCE perfusion and spectroscopy. The authors report the highest diagnostic accuracy to discriminate GBM and TRC for MR spectroscopy followed by MR perfusion. Yet, the authors proclaim various limitations of spectroscopy in the clinical routine, e.g., a large voxel size, long scanning times, and the technical challenges of this sequence [20]. Thus, in agreement with a recent survey on glioma imaging [33], use of spectroscopy is very limited in the clinical routine. In contrast, MR perfusion is widely used and established with excellent sensitivity and specificity [20]. In accordance, in our series minimum, mean, and maximum CBV and all CBV ratios showed significantly higher values in GBM than TRC, allowing the correct radiologic classification in 68–88% of included patients.

There are many different approaches for measuring CBV in DSC images. For example, Cha et al. analyzed CBV histograms, contouring the whole contrasting lesion. Their approach of comparing the histograms of an initial and a follow-up MRI showed a good sensitivity and specificity for discrimination of recurrent GBM and pseudoprogression via CBV with 81.8% and 83.3%, respectively [34]. Yet, their study is limited by the need of two consecutive MRI data sets with the consequent delay of a possible treatment of patients with recurrent tumors. Similar to our algorithm, Di Costanzo et al. also used a ratio between CBV in new, contrast-enhancing lesions and the contralateral healthy white matter, reporting a diagnostic accuracy of 86.2% for differentiation of GBM and TRC. Yet, the authors did not recommend a cut-off value for the parameter [35]. In a comparable approach, Kong et al. report a ratio_CBV_ cut-off of 1.47, reaching a sensitivity of 81.5% and a specificity of only 77.8% [36]. In contrast, the application of our multiparametric model based on minimum, mean, and maximum CBV in combination with minimum, mean and maximum ratio_CBV_ allows the correct identification of 94% of recurrent GBM. Thus, our algorithm—that yields convenient and rapid use in the clinical routine—may be a more robust approach for a reliable detection of recurrent tumor.

In van Dijken et al.’s meta-analysis, ADC imaging showed the lowest diagnostic accuracy among the included advanced MRI techniques to discriminate recurrent high-grade glioma and TRC [20]. In this context, Hygino et al. concluded that DWI does not provide sufficient information for differential diagnosis between pseudoprogression and true tumor progression [37]. Furthermore, in their review regarding diagnosis and treatment of pseudoprogression, radiation necrosis and brain tumor recurrence Parvez et al. state, that “reported ADC values are inconsistent and likely represent technical factors” in several studies [23]. Accordingly, in our study, ADC did not allow the reliable differentiation of recurrent GBM and TRC, with both groups showing decreased ADC values due to necrosis, intralesional hemorrhage, or formation of fibrosis [20]. Furthermore, selective measurement in the contrast-enhancing part of the lesion with the highest CBV did not improve the diagnostic power of ADC. However, in a small cohort of 20 patients, Song et al. tested ADC histogram analysis and showed the 5th percentile value of the cumulative ADC histograms as a promising parameter for identification of true progression in glioblastoma patients [38]. Further studies with larger cohorts are needed to verify their findings. The authors also had to transfer their MR imaging data to an external personal computer for analysis with a third-party software. Thus, our approach implemented in the clinical workflow might be more applicable.

Comparable to our approach, Wang et al. used DSC perfusion imaging in combination with DTI imaging in a multimodal model to differentiate true progression from mixed response/pseudoprogression in GBM patients. Based on a cohort of 41 patients, their multimodal model allowed the correct differentiation of these entities with 78% accuracy [24]. However, as the author’s analysis is based on time-consuming DTI imaging (reported acquisition time = 8 min) and requires two hours of post-processing, our model based on DSC imaging (acquisition time = 1:48 min), requiring 4 min for post-processing and yielding 94% accuracy for tumor identification, should be the favorable approach for routine clinical application. In a similar study, Prager et al. tested DWI and DSC perfusion to differentiate treatment-related effects (*n* = 10, DSC perfusion data available in only 4 patients) and recurrent high-grade gliomas (mixed WHO grades 3 and 4). Drawing a total of five ROIs in each ADC and CBV map, the authors report higher diffusion and lower perfusion in treatment-related changes [39]. While we cannot reproduce their ADC results (which might be due to our strict inclusion of only WHO grade 4 glioblastoma), Prager et al.’s CBV results are in accordance with our findings of decreased CBV in TRC.

Another approach for differentiation of recurrent GBM and TRC is the application of deep learning models. In a recent study, Akbari et al. used quantitative machine learning on a cohort of 40 patients to extract image features of conventional MRI sequences in combination with DTI and DSC data to differentiate both entities. The authors report a good accuracy for their model of 75% in their interinstitutional control cohort [22]. These are promising approaches; however, deep learning models are currently not applicable in the clinical routine due to extensive and time-consuming post-processing. The same limitation applies to radiomic approaches, although several studies showed promising results. For example, Kim et al. comprised 12 radiomic features (3 from conventional, 2 from diffusion, and 7 from perfusion MRI) in a model with a sensitivity of 91.4% and specificity of 76.9% [40]. In a similar approach, Elshafeey et al. boosted the accuracy of their radiomic model for identification of pseudoprogression in glioblastoma to 90.82% by combining 60 features of Ktrans and rCBV [41]. In this context, the combination of several three-dimensional shape features in a multiparametric model also enabled differentiation of true progression and pseudoprogression [42]. Thus, patients with glioblastoma might benefit from the future introduction of radiomic models to the clinical routine. Nevertheless, neuroimaging has general limitations regarding imaging of recurrent glioblastoma as it is well known that this tumor shows aggressive invasion into the surrounding tissue that is not definable in pre/post-contrast MRI and that extends into normal appearing parenchyma in MRI. This complicates total tumor resection and drastically limits overall survival [43,44,45].

This study has some limitations: First, the relatively low number of 34 included patients. Second, the retrospective analysis of the MRI data. Third, the possibility of overfitting our data in the multiparametric model. Thus, further prospective studies with larger patient cohorts are needed to verify the diagnostic strength of the proposed cut-off CBV and ratio_CBV_ values and our multiparametric model in the clinical routine. Fourth, the low number of histopathological proven TRC, as most patients with a new contrast-enhancing lesion after multimodal GBM treatment and suspected TRC will first receive a short-term MRI follow-up to exclude recurrent tumors in order to avoid possible biopsy-related complications and to decrease morbidity and mortality.

## 5. Conclusions

MRI DSC perfusion imaging allows good differentiation of recurrent GBM and TRC in our cohort. In contrast, neither selective nor unselective ADC enables discrimination of both entities. By combining minimum, mean, and maximum CBV with minimum, mean, and maximum ratio_CBV_, a multiparametric model improved identification of recurrent tumors surpassing the single-parameter approaches and reached 90% correct classifications.

## Figures and Tables

**Table 1:** Selected MRI sequence parameters for 1.5 T and 3 T MRI.

1.5 T	Contrast-Enhanced T1 MP-RAGE	DWI	DSC Perfusion with Leakage Correction
TR (ms)	2200	7600	2010
TE (ms)	2.67	86	30
flip angle (°)	8	-	90
FOV (mm^2^)	250	230	230
matrix (pixel)	256 × 256	324 × 372	128 × 128
voxel size (mm)	1.0 × 1.0 × 1.0	1.2 × 1.2 × 5	1.8 × 1.8 × 3
acquisition time (min)	4:59	1:25	1:48
**3 T**			
TR (ms)	1900	6600	1840
TE (ms)	3.16	95	32
flip angle (°)	9	-	90
FOV (mm^2^)	270	230	230
matrix (pixel)	320 × 320	180 × 180	128 × 128
voxel size (mm)	0.8 × 0.8 × 0.8	1.3 × 1.3 × 3	1.8 × 1.8 × 3
acquisition time (min)	4:09	1:41	1:39

DWI = diffusion weighted imaging; DSC = dynamic susceptibility contrast; TR = time to repeat; TE = time to echo; FOV = field of view.

**Table 2:** Patient data.

No.	Age	Gender	Tumor Resection (Month/Year)	Resection Status	IDH1 Mutation	MGMT Promotor Methylation	Radiotherapy (Month/Year)	Occurrence of New Contrast-Enhancing Lesion	Period from Radiotherapy to Occurrence of New Contrast-Enhancing Lesion (months)
glioblastoma									
**1**	40	male	01/2015	R0	unknown	methylation	02/2015–03/2015	10/2015	7
**2**	46	male	11/2012	R0	unknown	unknown	12/2012–02/2013	01/2014	11
**3**	55	male	09/2013	R0	no	unknown	11/2013–12/2013	06/2016	30
**4**	57	male	07/2016	R0	no	no methylation	08/2016–09/2016	09/2017	12
**5**	57	male	03/2017	R0	no	unknown	04/2017–06/2017	10/2017	4
**6**	59	male	02/2018	R0	no	no methylation	03/2018–05/2018	11/2018	6
**7**	60	male	03/2014	R0	no	unknown	11/2014–12/2014	05/2018	41
**8**	60	male	02/2014	R0	no	unknown	03/2014–05/2014	01/2019	56
**9**	61	female	11/2015	R2	no	unknown	11/2015–01/2016	12/2016	11
**10**	61	female	02/2013	R0	no	methylation	03/2013–04/2013	05/2016	37
**11**	64	male	01/2015	R2	no	unknown	02/2015–03/2015	06/2016	15
**12**	67	male	08/2018	R0	no	methylation	08/2018–09/2018	10/2018	1
**13**	69	female	01/2014	R0	unknown	unknown	03/2014–04/2014	01/2015	9
**14**	73	male	12/2016	R0	no	unknown	01/2017–02/2017	08/2017	6
**15**	73	male	10/2015	R0	no	unknown	11/2015–12/2015	06/2017	18
**16**	74	male	07/2018	R0	no	no methylation	07/2018–09/2018	04/2019	7
**17**	80	male	12/2013	R0	no	unknown	02/2014–03/2014	10/2015	19
treatment-related changes									
**1**	39	female	05/2015	R0	unknown	unknown	06/2015–07/2015	01/2018	30
**2**	40	male	06/2016	R0	mutation	unknown	07/2016–08/2016	03/2018	19
**3**	43	male	07/2010	R0	mutation	methylation	07/2010–09/2010; 11/2014	05/2016	18
**4**	45	male	03/2007	R0	mutation	unknown	04/2007–06/2007; 09/2015	07/2016	10
**5**	53	male	11/2011	R2	mutation	unknown	06/2015–08/2015	10/2015	2
**6**	53	male	09/2012	R0	unknown	unknown	10/2012–11/2012	04/2017	53
**7**	57	female	09/2012	R0	unknown	unknown	10/2012–11/2012	12/2012	1
**8**	63	female	05/2017	R0	no	unknown	06/2017–07/2017	02/2018	7
**9**	64	female	10/2006	R2	unknown	unknown	11/2006–12/2006	10/2018	142
**10**	65	male	03/2018	R0	no	unknown	04/2018–05/2018	07/2018	2
**11**	66	male	06/2017	R0	no	unknown	07/2017–08/2017	12/2018	16
**12**	68	female	08/2014	R0	no	unknown	09/2014–11/2014	03/2017	28
**13**	76	male	05/2018	R0	no	unknown	07/2018–08/2018	10/2018	2
**14**	80	male	12/2016	R0	no	methylation	01/2017–02/2017	06/2017	4
**15**	82	female	10/2017	R0	no	no methylation	11/2017–12/2017	05/2018	5
**16**	85	male	05/2016	R0	no	methylation	06/2016–07/2016	09/2016	2
**17**	87	female	10/2013	R0	unknown	unknown	11/2013–12/2013	01/2015	13
IDH1 = isocitrate dehydrogenase 1; MGMT = O6-methylguanine-DNA methyltransferase									

**Table 3:** Selective and unselective ADC values in GBM versus TRC.

	GBM	TRC	*p*-Value	AUC
selective minimum ADC	955.0 ± 57.6	997.5 ± 75.8	0.5861	0.557
selective maximum ADC	1620 ± 84.9	1546 ± 93.8	0.5644	0.554
selective mean ADC	1254 ± 42	1261 ± 73.7	0.9326	0.512
unselective minimum ADC	696.7 ± 75.8	820.9 ± 63.1	0.2167	0.614
unselective maximum ADC	2257 ± 94.2	2072 ± 133.5	0.2054	0.630
unselective mean ADC	1335 ± 39.9	1355 ± 76.6	0.8205	0.523

ADC = apparent diffusion coefficient; GBM = glioblastoma; TRC = treatment-related changes; AUC = area under the curve.

**Table 4:** CBV values in GBM versus TRC.

	GBM	TRC	*p*-Value	AUC
minimum CBV_lesion_	57.7 ± 80.6	25.3 ± 59.5	0.0391 (*)	0.706
mean CBV_lesion_	260.6 ± 150	83.5 ± 64	0.0003 (***)	0.851
maximum CBV_lesion_	698 ± 406.5	202.9 ± 105.9	<0.0001 (****)	0.8737
ratio_CBV minimum_	1.6 ± 2.2	0.55 ± 0.88	0.0169 (*)	0.737
ratio_CBV mean_	4.3 ± 2.6	1.4 ± 0.9	<0.0001 (****)	0.917
ratio_CBV maximum_	7.3 ± 3.9	2.3 ± 1.4	<0.0001 (****)	0.917
minimum CBV_unaffected_	36.7 ± 26.9	44.1 ± 49.4	0.8717	0.517
mean CBV_unaffected_	62.9 ± 7.6	72.2 ± 14.3	0.7660	0.531
maximum CBV_unaffected_	94.9 ± 40.1	108.6 ± 76.5	0.9927	0.502

CBV = cerebral blood volume; GBM = glioblastoma; TRC = treatment-related changes; AUC = area under the curve. *p*-values less than 0.05 are marked with “*”, less than 0.001 with “***” and less than 0.0001 with “****”.

**Table 5:** Classification of patients with recurrent GBM and TRC using single- and multiparametric models.

	Cut-off	Correctly Classified (%)	Identified Recurrent GBM (%)	Identified TRC (%)
CBV_minimum_	8.5	68	12 of 17 (71%)	11 of 17 (65%)
CBV_mean_	116.5	82	14 of 17 (82%)	14 of 17 (82%)
CBV_maximum_	327	85	14 of 17 (82%)	15 of 17 (88%)
ratio_CBV minimum_	0.17	74	15 of 17 (88%)	10 of 17 (59%)
ratio_CBV mean_	2.26	85	15 of 17 (88%)	14 of 17 (82%)
ratio_CBV maximum_	3.82	88	15 of 17 (88%)	15 of 17 (88%)
multiparametric model	-	88	16 of 17 (94%)	14 of 17 (82%)

CBV = cerebral blood volume; GBM = glioblastoma; TRC = treatment-related changes.

## Data Availability

The data presented in this study are available on request from the corresponding author. The data are not publicly available due privacy restrictions.
